# Possible COVID-19 Maternal-to-Neonate Vertical Transmission in a Case of Early Neonatal Infection

**DOI:** 10.7759/cureus.27141

**Published:** 2022-07-22

**Authors:** Trupti Pandit, Ramesh Pandit, Keshav Bhattar

**Affiliations:** 1 Pediatrics/Hospital Medicine, Nemours Children's Health and Inspira Medical Center, Glen Mills, USA; 2 Medicine, Independent Researcher, Philadelphia, USA; 3 Hospital Medicine, University of Pennsylvania/Chester County Hospital, Philadelphia, USA; 4 Internal Medicine, Dr. Sampurnanand (SN) Medical College, Jodhpur, IND

**Keywords:** covid vaccine in pregnancy, covid-19, covid-19 prevention, maternal-child health, neonatal respiratory distress, covid-19 vaccine, vertical covid-19 transmission, neonatal covid-19 infection, covid-19 in pregnancy, congenital covid-19

## Abstract

Coronavirus disease 2019 (COVID-19) is a disease caused by a novel strain of coronavirus and has resulted in a global pandemic. Information regarding the COVID-19 pathophysiology and its long-term impacts on humans is yet to be found. The knowledge about the COVID -19 infection's effects on the fetus is limited. The maternal to fetal transmission during various trimesters is not adequately studied. We present a case concerning maternal-to-fetal vertical transmission focusing on congenital infection.

## Introduction

The coronavirus disease 2019 (COVID-19) was declared a global pandemic by the World Health Organization (WHO) on March 11, 2020. It has been observed that pregnant women are at a higher risk for developing severe COVID-19 than the general population [1]. Based on pre-existing data, fetal distress, abortion, preterm delivery, and stillbirth are the suggested risks for pregnant women with COVID-19 [1]. The rising number of cases of infected pregnant women and newborns raised concern and underlined the necessity to find evidence for vertical transmission, which is the transmission of severe acute respiratory syndrome coronavirus 2 (SARS-CoV-2) from mother to fetus.

## Case presentation

A 29-year-old female, gravida 5, parity 4, delivered a male neonate (patient) vaginally, after spontaneous labor, at 40 weeks and four days of pregnancy. Preventive screening of the mother during pregnancy included a negative group B streptococcus test, unremarkable ultrasound evaluation, and a normal glucose tolerance test. The mother’s prenatal tests for syphilis, hepatitis B, and HIV were negative. Amniotic fluid was noted to have thick meconium. The Apgar score was noted to be eight at one minute and nine at five minutes. The neonate weighed 3270 gm, which was at the 15th percentile, length was 52 cm (51^st^ percentile), and head circumference was 34 cm (15t^h^ percentile). The neonate was appropriate for gestational age.

At 24 hours of life, the neonate was noted to have an increased respiratory rate at 80/min with subcostal and intercostal retractions. Oxygen saturation was noted to be 78% on room air.

Investigations

**Figure 1:**
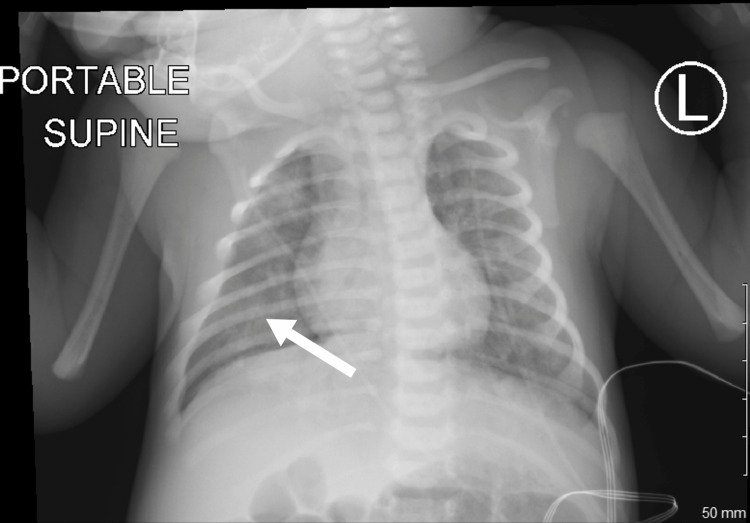
Chest X-ray on day of life 2, significant for bilateral infiltrates, right (white arrow) greater than left

**Figure 2:**
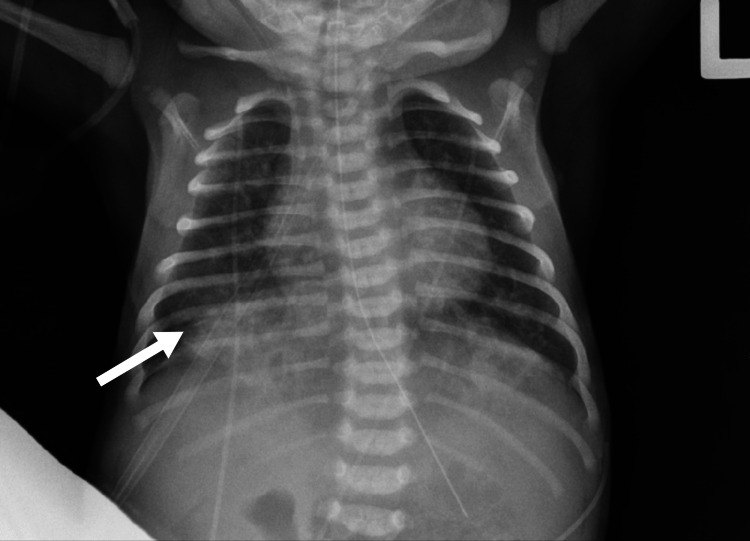
Chest X-ray on day of life 3: right lung base infiltrates increased (white arrow)

Chest X-ray (CXR) was significant for bilateral infiltrates, as shown in Figure 1 and Figure 2.

Lab results were as detailed in Table 1.

**Table 1:** Lab result findings BUN: blood urea nitrogen; pCO2: partial pressure of carbon dioxide; pO2: partial pressure of oxygen

Lab Test	Results	Normal Range
White blood cell (K/l)	25.6	At Birth: 9-30, Age 12 hrs: 13-38, Age 24 hrs: 9.4-34
Platelet (K/l)	253	150-400
Neutrophils (%)	83%	20.2-46.2
Band	2%	<20 % of PMN
Reticulocyte count (%)	1.7	3.47-5.40
Sodium (mmol/L)	141	132-142
Potassium (mmol/L)	3.8	5.0-7.5
Bicarb (mmol/L)	27	22.0-26.0
Calcium (mg/dL)	8.3	7.8-11.3
BUN (mg/dL)	14	5-17
Creatinine (mg/dL)	0.8	0.6-1.1
Arterial Blood Gas Test		
pH	7.38	7.27-7.47
pCO2 (mmHg)	36.1	29-45
pO2 (mmHg)	46	55-106
Oxygen saturation (%)	90	85-90
Direct Coombs test	Positive	Negative
Neonate's blood type	A+	
Mother‘s blood type	O+	

As part of the admission requirement, the mother had a nasal swab for COVID-19 screening though she didn't report any COVID-19-related symptoms. The mother's routine COVID-19 polymerase chain reaction (PCR) result was reported to be positive 24 hours post-delivery. Around 24 hours of life, the neonate had tachypnea, so was separated from his mother and taken to the neonatal intensive care unit (NICU) for further care. The neonate had a COVID-19 nasal swab PCR test on the second day of life and was reported to be positive. Repeat COVID-19 nasal swab PCR tests collected on the day of life 5 and 10 were also positive. The blood culture was done to rule out bacterial infection and was reported negative after five days. Post-discharge, he did not have a repeat COVID test during the neonatal period, as he was clinically doing well.

Treatment

The neonate was started on oxygen support, initially on 3L of oxygen via nasal cannula, and was transferred to the neonatal intensive care unit (NICU) for further intensive care needs, and was placed on high-flow nasal cannula 3L with a fraction of inspired oxygen (FiO2) of 50%, with an improvement in oxygen saturation above 94%. The neonate’s blood type was reported to be A+. Direct Coomb’s test was positive. The bilirubin level was in a high-risk zone, per the bilirubin nomogram. Per the American Academy of Pediatrics (AAP) phototherapy guidelines, the neonate was started on phototherapy [2]. Follow-up bilirubin levels are reported in Table 2. Phototherapy was continued for a duration of one and a half days. After discontinuing phototherapy, the follow-up bilirubin level was 9.5 at 77 hrs in the low-risk zone.

**Table 2:** Bilirubin levels

Time since birth (in hours)	12 hours	23 hours	36 hours	54 hours	77 hours
Bilirubin (mg/dl)	9.5	10.8	10.5	10.6	9.5
Risk Zone as per The Bilirubin Nomogram [2]	High	High	High Intermediate	Low Intermediate	Low Risk

He was weaned off oxygen to room air on his 10^th^ day of life. He received IV ampicillin and gentamicin antibiotics due to suspicion of meconium pneumonitis vs. pneumonia. After five days, the blood culture was reported negative, so antibiotics were stopped. The neonate was tolerating oral feeds well. He recovered well and was discharged home. At one year, the patient was growing appropriately. There were no development delays noted.

## Discussion

Initial Chinese studies ruled out the vertical transmission of the SARS-CoV2 virus based on two key findings: 1. amniotic fluid, vaginal mucus, placenta, umbilical cord, cord blood, and neonatal stool specimens tested negative for the virus [3-4]; and 2. There were no reports of vertical transmission during the Severe Acute Respiratory Syndrome (SARS) and Middle East Respiratory Syndrome (MERS) outbreaks caused by genetically similar coronaviruses [5]. However, case reports from 2020 showed that despite negative virology testing, COVID-19-specific immunoglobulin M (IgM) antibodies (which cannot cross the placenta due to their larger size) were found in seven babies [6].

Studies across many countries have reported various incidences or suspicion of perinatal COVID infection (ranging from 0.5 to 3.5%) [1,7]. However, most neonate illnesses are transmitted after birth, and outcomes are predominantly favorable. Prematurity and comorbidities have been implicated in most poor neonatal outcomes. Regardless, neonatal COVID-19 infections have been noted to increase the rate of NICU admissions and pose a substantial burden on health infrastructure [8]. More recent studies have identified the cell membrane-associated angiotensin-converting enzyme 2 (ACE-2) and the transmembrane protease serine 2 (TMPRSS2) required for SARS COV-2 transmission in placental tissue and utero transmission from the transplacental transmission can occur from any inflammatory process causing damage to the placenta [9]. Only mothers with severe COVID-19 illness or high blood viral levels have transvaginal and amniotic transmission [10].

Neonates and infants are less susceptible to symptomatic SARS-CoV-2 infection than adults and have a milder sickness with a lower mortality rate [11-12]. Potential explanations include immature immune systems possibly resulting in a less destructive cytokine reaction, transplacental transfer of maternal antibodies during the third trimester, and ACE-2 receptors being either under-expressed or functionally immature. The neonatal clinical presentation differs from that in older infants and adults, with gastrointestinal symptoms and poor feeding being more common [13].

Greater expression of ACE-2 receptors in SARS-COV-2-induced hepatocyte and cholangiocyte injury result in impaired uptake and impaired excretion of bilirubin [14]. Pneumonitis and ongoing inflammation can cause hyperbilirubinemia through impaired hepatocyte nutrition and toxemia. This newborn had pathological jaundice (appearing within the first 24 hours of life, with a rise in bilirubin of > 0.2 mg/dL/hr and requiring treatment). Direct Coombs test was positive, most likely due to the mismatch in the blood types: A+ in the newborn and O+ in the mother. We hypothesize that the primary cause of this neonate’s jaundice was hemolysis due to ABO incompatibility, but the concurrent COVID-19 viremia and inflammation may have contributed via the mechanisms mentioned above.

Our neonate had good Apgar scores at birth (eight at one minute and nine at five minutes). The onset of respiratory distress was around 24 hours after delivery. The diagnosis of meconium aspiration syndrome (MAS) usually presents with respiratory distress hours after birth, with non-reassuring Apgar scores and chest imaging showing hyperinflated lungs with flattened hemidiaphragm secondary to distal small airway obstruction and gas trapping; and asymmetric patchy pulmonary opacities due to subsegmental atelectasis with or without pleural effusions, pneumothorax, or pneumomediastinum [15]. However, these findings are neither sensitive nor specific to MAS, and chemical pneumonitis produced from MAS can have imaging similar to those found in our neonate. Most COVID-19 neonatal infections tend to appear early (within the first 48 hours) [16], similar to our neonate. However, there are other case reports where neonates developed delayed (>72 hours) hyperbilirubinemia and pneumonia or pneumonitis, with some cases progressing to sepsis, requiring more than two weeks of hospitalization [17]. The neonates were noted to have a high incidence of hyperbilirubinemia with maternal COVID-19 infection [18], similar to our neonate.

According to the INTERCOVID Multinational study, the presence of COVID-19 infection is associated with significantly increased neonatal complications and maternal morbidity and mortality [19]. Managing COVID-19-infected newborns < 28 days can be challenging, as no approved antiviral medications are available. Antiretrovirals such as remdesivir have emergency use authorization (EUA) for infants > 28 days and > 3.5 kg [20], while Paxlovid and monoclonal antibodies have EUA for children 12 years and older [21]. A double-blind study comparing ivermectin vs. placebo showed decreased viral load in COVID-19 adult patients, but the use of the same has not been evaluated adequately with the associated side effects [22-23]. The Centers for Disease Control and Prevention (CDC) has declared vaccination for COVID-19 safe for pregnant women, and it is prudent to vaccinate expecting mothers in the last trimester so the fetus can have the passive transfer of protective antibodies, given the information that vaccine-based immune response is maximum for three months duration [24]. For neonates to benefit from IgG antibodies from the mother transplacentally, maternal vaccination is recommended at least four weeks before the expected delivery date [25].

## Conclusions

From this case, we think that COVID-19 might have vertical transmission and can result in fetal or newborn infection. Possible congenital COVID-19 newborn infection may lead to pneumonia, hypoxia, and prolonged length of stay, as seen in our case. The congenital infection complications resulting from maternal COVID-19 infection still need further long-term studies. Though the prognosis for neonatal COVID-19 infection is relatively favorable, preventing both vertical and horizontal transmissions is essential to reduce neonatal morbidities and length of hospital stay. The vaccination for COVID-19 is considered safe for pregnant women. Vaccinating expecting mothers in the last trimester and at least four weeks before the expected delivery date is important for neonates to receive adequate antibodies transplacentally.
